# “Sports Maternalism”, to Train and Take Care: Ethnographic Investigation in Baton Twirling Clubs

**DOI:** 10.3389/fspor.2020.556195

**Published:** 2021-01-06

**Authors:** Shia Manh Ly

**Affiliations:** ^1^Institut de Sociologie, Faculté des Lettres et Sciences Humaines, Université de Neuchâtel, Neuchâtel, Switzerland; ^2^Haute école de travail social et de la santé Lausanne HETSL – HES-SO, Lausanne, Switzerland

**Keywords:** ethnography, sports club, sociabilities, care, gender, sportification

## Abstract

This article is the result of an ethnographic work on baton twirling clubs in Switzerland: clubs with few members coming from a modest origin, offering a social and physical activity with little resonance, composed of children, and young girls. The supervision is mainly the responsibility of close volunteers: family members, friends or neighbors and, for the majority of them, women. It is therefore an environment where people know each other, where gestures of familiarity are the rule and where tensions may sometimes arise due to various conflicts of proximity. Baton twirling is based on a public display of participants and the competitive aspiration for a self-presentation that solicits feminine stereotypes. It shows sociabilities and socialities framed by gender and age relationships: within clubs, knowledge transmission and childcare are combined in women's practices. The relationships between women and children transcend learning relationships. These relationships, which go beyond a vertical transmission of knowledge, call for approaches inspired by the theories of *care*. What is the meaning of these relationships based on women's care from the point of view of sociality and in relation to the institution of sport? This is the main question that will be addressed here. Approaches of care emphasize accompaniment, maintenance. They seem to be a good way to identify the contours of a “sports maternalism” which makes such a commitment valid while at the same time conferring legitimacy on a sports practice that is poorly considered.

## Introduction

If the historiography of sport has for a long time largely marked out the themes of identities–be they political, cultural, social, ethnic or gender -, it has not shown the same interest in questions relating to the characteristics and determinations linked to age in sporting practices. In Switzerland, proportion of children and adolescents joining sports clubs is increasing, more than a third of active members are under 20 years of age and the greatest growth is seen among children (Marston Tallec, 2015) under 10 years of age (Lamprecht et al., 2017). Although well established, sports clubs have a lack of attention in terms of producing social identities.

While cultural practices and behaviors of children and adolescents have been extensively studied in relation to institutions such as family or school as places of socialization, only some research has dealt with leisure activities in the context of associative life. Sport clubs occupy the reflection in French-speaking sociology of sport for example in terms of response to social problems (Pantaléon, 2003; Gasparini and Vieille-Marchiset, 2008; Coignet, 2013). To sum up, sociology of sport addresses sports clubs generally following, regardless of their national focus, a vertical transmission of values approach. However, the interest of this article is to grasp the club from the perspective of associative socialities and sociabilities.

The subject of this article is the result of a sociological analysis that follows a 3-year ethnographic method in the framework of a thesis^1^. This thesis is devoted to baton twirling clubs in Switzerland, which are small clubs offering a social and physical activity with feminine connotation. The stereotypical but also outdated character of this activity makes it little known and undervalued^2^, contrary to football or tennis for example. These undervalued practices, by valuing their own codes and symbols, resist the cultural forms placed higher up in the hierarchy of social values and confront the cultural contempt they are likely to suffer (Radway, 1984; Brown, 1994) by the place they occupy, i.e., on the margins of “legitimate culture” (Bourdieu, 1979).

Clubs are mainly composed of girls aged between 5 and 20 years. The supervision is the responsibility of close volunteers: family members, friends or neighbors and, for the majority of them, women. It is therefore an environment where people know each other, where gestures of familiarity are the rule (kisses, hugs, confidences, jokes, etc.) and where tensions can sometimes arise due to various conflicts fueled by this great closeness (jealousy, slander, etc.).

Like any sport discipline, baton twirling is based on public exposure of the participants and the competitive aspiration to a self-presentation that solicits female stereotypes (pink bodysuit decorated with sequins, sexualized choreography, etc.). The baton twirling shows sociabilities (ways of living in society) and socialities (set of social bonds) woven by social relations of sex and age: within the clubs, transmission of knowledge and childcare are combined in women's practices.

These practices are invisible, as they are carried out by women and fall within the private sphere. What meaning then do these women give to their practices and how do they give them legitimacy? Questioning these practices from the angle of the notion of *care* from a feminist perspective, notably with the contributions of Laugier (2009) and Gilligan (2009), allows us to talk about the situations, commitments, and spaces occupied by women and the gestures associated with them. These care practices seem to be well-suited to identify the contours of a sports maternalism which, in addition to guiding the action of the instructors, makes such a commitment valid. At the same time, this sports maternalism confers legitimacy on a sports practice that is poorly considered.

## Care Approaches

The focus is on a popular and meaningless activity, baton twirling, by the image it reflects of an aesthetically traditional and conservative femininity, of an ordinary corporality (clubs choose to accept all bodies), but also of low valued cultural tastes (baton twirling is practiced on variety music by athletes with handmade personalized costumes).

If there is little interest in the activity, it is also because it is based on a form of sociality articulated around the poles of the feminine and the familial. Indeed, baton twirling is an activity that mobilizes women, mothers who care for girls, children. These women and girls, which are characterized by gender inequalities and female stereotypes that are poorly valued, are also people from modest backgrounds. The study thus brings together feminist approaches, particularly of care.

Care (Tronto, 2009; Molinier, 2013; Modak, 2015; Skeggs, 2015) is all the material and emotional practices involving support, guidance, assistance, etc. provided by women of lower social status. Historically, these practices have been perceived as private, because they are carried out by women and involve feelings. However, care contains a political dimension because it is public, as Sandra Laugier explains, by the fact that dependence and vulnerability “are features of everyone's condition” (Laugier, 2009). For Laugier, the ethics of care, by its concrete character, is a real activity, a work and an attitude, the “guiding thread” of humanity (Laugier, 2009). Care approaches are feminist. On this point, Laugier agrees with Giligan when she claims the ethics of care as a democracy without forms of intolerance such as patriarchy, sexism, racism, etc. (Laugier, 2009). The feminist ethics of care wants to detach itself from the gender dichotomy and articulate itself around democratic norms and values (Gilligan, 2009).

Typically, care to children is qualified as feminine and popular and revealing of an unequal distribution system. Susan B. Murray explains the difference between men's “primary” incomes and the “supplements” provided by women, which lead women workers to accept “emotional compensation” instead of financial rewards (Murray, 1996, p.372). Care also allows us to understand how these practices, which are denigrated because they are unregulated and based on a sociality of familiarity and love, are defined and categorized by committed actors.

## Questions

What is the meaning of relationships based on the care taken by women from the point of view of sociality and in relation to the sports institution in the case of baton twirling clubs? How are the values of closeness and support defended or even claimed? How do they stand out in the ordinary life of clubs? Or how do the status of mothers who look after children and the status of instructors who train young sportswomen combine? These are the questions that will be addressed in this article with the help of excerpts from observations, interviews and field notes. The aim is to grasp how a “sports maternalism” is constructed and how it directs and maintains a fragile sport (to make the practice “pleasant” in a “warm” environment), whose legitimizing horizon lies in a threatening sportification.

## Methods

### Procedure

The sociological analysis carried out follows an ethnographic, inductive, and interactionist method. The aim is to analyze the practical action, interactions, categories and interpretations of the actors on their commitment and the meaning they give to it. Within the framework of this research, for the duration of 3 years, 35 observations were carried out to capture the interactions between adults, between adults and children and between children, during training sessions, competitions, general assemblies, and end-of-season parties. Sixteen collective interviews were conducted by a member of the research team with groups of two to four athletes, selected according to their affinities. They have been provided with a parental agreement document to fill in beforehand. Interviews with the children were carried out in groups in order to help them to speak more freely (Golay and Malatesta, 2012). Twenty interviews were also set up with members of the coaching staff, clubs committees and the federation. For the observations, the aim was to follow a precise framework in order to target the research objectives quickly. The interviews followed the same process with open-ended questions also grouped by theme (choice of sport, entry into the “career,” etc.). Documentary analysis, press articles, official documents of the clubs and the federation, etc., were also used, which made it possible to understand the functioning of the institution and the processes of visibility of the clubs.

### Participants

The chosen fields are three baton twirling clubs in French-speaking cantons of Switzerland. Close to gymnastics and ice skating, baton twirling differs from majorettes^3^ by its sportification and competitiveness. It is part of Swiss Olympic, the umbrella organization of Swiss sports federations, and is recognized by Jeunesse + Sport, the Swiss program for the promotion of sport among young people in the discipline of gymnastics and dance. The Swiss baton twirling club federation FSTB has around 300 athletes in 12 clubs and is therefore one of the “small” federations (Lamprecht et al., 2017).

Two of the clubs are located in cities (one of more than 200'000 inhabitants and the other of more than 20'000), and training takes place in gymnasiums (school, sports complex, cultural complex). One of them is on the outskirts (village of about 5,000 inhabitants) and training always takes place in the same school.

One of the urban clubs is the result of a split of majorettes troops and has a family management, the grandmother being responsible for the athletes, the mother being president of the club and the daughter, a former athlete, being the technical manager and in charge of the instructors. Nevertheless, kinship ties between adult club members and athletes are almost non-existent in contrast to the second club located in the city or the village club.

Each club is composed of about twenty athletes, a few coaches and a committee (often with a president, secretary and treasurer, who are also sometimes coaches). In the committees, there are many former athletes, or mothers/fathers of athletes who fill in positions because of a lack of people volunteering. The same goes for coaching, where parents, brothers and sisters are brought in, and sometimes find themselves coaching members of their own family. In many cases, adults wear two hats, as club president and instructor, or technical manager and instructor. Moreover, recruitment is done by word of mouth. Athletes are often neighbors, classmates, brothers, sisters, etc. They are between 5 and 20 years old and have origins that can be qualified as popular, by sociabilities and tastes.

## Results

The examples mobilized have been classified according to defined thematic categories. These thematic categories are specific to the approach of analytical induction, i.e., they are derived from the field through interviews and observations. They answer the following question: What is fundamental in this word or gesture? (Paillé and Mucchielli, 2012) To do this, it is necessary to proceed in two steps, first to identify the significant ideas and then to categorize them. This is how the raw data is processed (Negura, 2006). These thematic categories include the relationship to health and pain, to learning through caring and listening, to managing disappointment and attachment to the athlete, and to managing sportification. They seem to be the most relevant for understanding the articulation between care, sociality, and sports institution. The following are excerpts illustrating these care practices in both training and competition.

To get to the heart of the matter, the first excerpt summarizes the close relationship between athletes and instructors during training sessions, through the nicknames given as well as the tactile behavior:

The coach Christelle^4^ calls [the beginners] “come over here sweeties.” She gets closer to the stage of the room, looking at a sheet of paper, and shows something to the girl who was drinking “It's beautiful, isn't it? These little hands, these little feet.” They were looking at a figure cut from yellow paper. Then, the coach takes the girl in her arms and smiles at me before crossing the room. (Observation, 16.11.15)

### Relation to Health and Pain

Throughout the sport season, there is concern for the athlete and her health. The instructors scold the girls when they go outside without a coat during the cold season, make sure that they breathe and eat well-before a performance. There is in the following excerpt another type of concern, at first sight a desire to preserve the athlete's privacy:

When Aline [senior athlete] finishes her choreography, Josette [coach and club's president] makes her understand that her performance was average. Aline is about to cry. Josette takes her in her arms. They walk together and go outside the gym hall. Josette announces to the other girls, “time for a break, I'll be back” […] Tania [coach] is about to leave. […] Before she goes, she asks Josette, who comes back alone, if Aline is okay. Josette doesn't answer the question. “It's something else,” she says. (Observation, 27.01.16)

The examples given above illustrate care as they relate to the “physical” aspect, the body but also the mental of the athletes. The relationship to health and pain is an eloquent theme about how adults care for the well-being of children as if they were their own. Sorignet (2006) shows that pain is inseparable from the practice of professional dancers, perceiving it as something to be overcome and necessary for progression. Here, the logic is different. The observations have shown that instructors never “force” athletes to continue when they feel physical pain. On the contrary, they are invited to rest. When possible, the instructors arrange to treat them:

Lena [junior athlete]'s foot still hurts. She stays aside and Carla [coach] comes to see her, she quickly massages her foot. (Observation, 23.11.15)

### Caring and Listening, Keys to Learning

Learning is always done with benevolence. The instructors never go against the abilities and desires of the athletes, as these excerpts underline:

Eleonor [invited coach] shows Lucia [junior athlete] a maneuver “you don't have to throw yourself.” Lucia tries. Eleonor corrects, “stretch your legs a bit, d'you think you can do that?” […] Lucia smiles and says “no.” Eleonor answers, “I won't force you to do that.” (Observation, 29.10.15)

I ask Chloé [coach] if the athletes train hard before the championships. Chloé says, “they are kids, they've got their own rhythm.” (Observation, 21.01.16)

At the end of the performance, Valentina [junior athlete] does a back walkover [acrobatic maneuver] and falls back on her feet. Tania [coach] concludes, ≪Vale, if the back walkover doesn't work, we won't do it.≫ (Observation, 27.01.16)

Murray explains the extension from maternal qualities to professionalization, “perceiving women childcare workers as mothers and conceptualizing their work as mothering, for instance, overshadows other possible perceptions of them as teachers and professionals.” (Murray, p.383). In these examples, we can see that for the choreographies and the preparations for competitions, the instructors remain attentive to the children. We can find in coaches an extension of the so-called “maternal qualities.”

### Managing Disappointment and Commitment to the Athlete

The competitive moments are a major stress for athletes who sometimes perform below their expectations. They train the whole season to present a choreography of <2 min, which can generate some frustration. These disappointments often go hand in hand with sadness or anger. The athletes see the chance of qualifying or obtaining a medal disappear. The excerpt below shows how hard it is to manage these situations:

“- How do you feel about the athletes not wanting to disappoint you?”

“- Sometimes it's hard to handle because no matter what happens, they give the best of themselves. […] I've seen that with Béatrice [junior athlete] last year, when she did her artistic performance. […] She wasn't selected. It was very hard to see her cry […]. But despite everything, I was very proud of her. She gave the best of herself and that's it. It's very hard to handle this for her, but for me, too […] And at our ages, we learn to relativize, to let things go. But not at their ages […] Because after that, she lost her motivation, she thought that her skills sucked. […] I can't let them down because I'm affected. If they are too sensitive, I'll be too sensitive too and I'll want to protect them. And we'll find ourselves in a vicious circle.” (Interview, 20.09.17)

This excerpt illustrates the empathy felt by the instructors. They not only follow their performances closely, but also by share with them their disappointment as this coach explains. We note that she nevertheless speaks of a “vicious circle” which may imply a willingness not to get too involved in the intimate life of the athlete despite the desire to take care of the vulnerable child in front of her.

### Managing Sportification

Baton twirling faces the logic of “sportification,” a social and institutional process aimed at promoting an edutainment of sports status (Parlebas, 1999, p.379). It is illustrated here by an increase in requirements and, consequently, a pronounced individual selection of athletes. Threatening sportification is the most revealing dimension of these care relationships. Indeed, there is a willingness on the part of instructors not to “rush” the athlete and to make the activity “good.” For example, during the exploratory interview, a club president stated that “elite is good, but it punishes others.” She added that “competitions should be open to everyone” but that “now they [Federation members] are closing all the doors.” For her, “we do baton twirling because we like it” (Josette, exploratory interview, 11.06.15). The subject came back during a training session:

Josette [coach and club's president] says that sometimes, there is too much pressure and the girls stop their activity. I ask if it has already happened in her club. Josette answers that “we make sure that they don't go that far.” […]She explains that the Haldi's cup was made for the little ones but now, you have to pass some qualifying exams, this “stuff.” […] Sometimes the juniors can't participate at all. She shows me a girl with orange pants, walking in front of us, “for example, she has to pass this stuff and for the moment, she's left out.” (Observation, 25.11.15).

There has been a mobilization in the name of the principle of integration of all sportswomen notably through a collective letter addressed to the federation, which resulted in a positive outcome, with the opening of a new, less restrictive category during the various rounds of the Swiss championship.

## Discussion

The purpose of this article was to understand the relationships and meanings that are played out within small clubs and in relation to the sports institution. The previous excerpts illustrate in a concrete way the stakes around this sports maternalism from the point of view of care, a notion that makes it possible not to lock the actors on the field into a closed universe but to emphasize what this type of relationship to the other means.

It is possible to find these words and examples in other clubs. It is not characteristic or specific to the baton twirling. However, this approach from the angle of care is essential to the analysis of these practices and the places in which they take shape. The examples make particular sense when clubs face sportification. The practices that take place within these small communities can then be read as a claim for a better place for sportspeople who do not meet the criteria of excellence. Sportification provides an understanding of how clubs “reinterpret” their practice by intensifying a “common membership” based on caring relationships, without paying attention to each other's abilities (Malatesta et al., 2014). We are moving away from judgement to create a more local “sports culture” (Malatesta et al., 2014).

In fact, we can see it in the mobilized excerpts touching different categories, in reactions and gestures, that mothers, sisters, etc. take on several roles such as instructor, animator, etc. but what deserves to be retained is that young athletes are not on the bangs of the phenomenon. Taking on the roles of friend or teammate, it goes without saying that they have above all their primary role: that of a child (Malatesta et al., 2014). This plurality of roles taken on explains the specificity of relationships and their meaning. It can be said that this sports maternalism is not an application of a “savoir-vivre.” It is a response to the message addressed by the sports world, which not only marginalizes the practice of baton twirling but which at the same time forces it to become sportified.

Analyzing clubs as places of socialities and sociabilities has relevance for research in sociology and development. This leads to a better knowledge in terms of organized leisure, all the more so when it has a low social and cultural value. It helps to understand associative engagement as well as the relationship to the public and democratic space, a relationship that reveals various issues, notably interests and power between social groups. Taking into consideration care and “sports maternalism” within the associative life also contributes to the understanding of the commitment of minority publics in society. This contribution thus has a resolutely political dimension. It offers knowledge on a theoretical and empirical level, but also on a methodological level insofar as it opens up the discussion on the modes of investigation of the involvement of minority groups in fragile organizations.

## Conclusion

We have seen through this article how care practices are at the base of baton twirling clubs through the different interactions that take place. Within the clubs, it is almost not possible to wear a unique hat, the status are combined. It is a question of caring relationships in the face of the ever-increasing sportification of baton twirling. The aim is to encourage girls to persevere and to “keep” them in a fragile sport. It is therefore a question of moving away from this logic of sportification imposed from “above,” in order to make the practice more enjoyable.

Above all, there is a strong will to preserve a solid being together, which translates into a “gift of self.” One takes care of an athlete as one would take care of one's own daughter. Care thus takes on its full meaning when talking about sports commitments, but also about promoting the activity and “fighting” against the institution. It allows us to understand the meaning of this care for others but also for ourselves since it is a human condition.

## Data Availability Statement

The raw data supporting the conclusions of this article will be made available by the authors, without undue reservation.

## Ethics Statement

No potentially identifiable images or data are included in this article.

## Author's Note

This article is the result of a sociological analysis that follows a three-year ethnographic method implemented in the framework of a thesis and of a research carried out by Division 1 of the Swiss National Science Foundation (SNSF) in collaboration with Thomas Zannin, Prof. Dominique Malatesta and Prof. Christophe Jaccoud.

## Author Contributions

The author confirms being the sole contributor of this work and has approved it for publication.

## Conflict of Interest

The author declares that the research was conducted in the absence of any commercial or financial relationships that could be construed as a potential conflict of interest.

## Footnotes

^1^This thesis is the result of research carried out by Division 1 of the Swiss National Science Foundation (SNSF) in collaboration with Thomas Zannin, Prof. Dominique Malatesta and Prof. Christophe Jaccoud. It was also financed within the framework of this project.

^2^This has repercussions with less infrastructure or reduced financial resources.

^3^Traditionally, majorettes troops are composed of girls. They dress in costume and wield a metal stick following a choreography during public parades. They are usually accompanied by marching bands.

^4^First names have been changed in order to preserve anonymity.

## References

[B1] Bourdieu P. (1979). La distinction. Critique Sociale du Jugement. Paris: Minuit.

[B2] Brown M. E. (1994). Soap Opera and Women's Talk. The Pleasure of Resistance. London: Sage.

[B3] Coignet B. (2013). Sport et Innovation Sociale. Des Associations Sportives en Mouvement Dans les Quartiers Populaires. Paris : L'Harmattan.

[B4] Gasparini W. Vieille-Marchiset G. (2008). Le Sport Dans les Quartiers. Pratiques sociales et politiques publiques. Paris : Presses Universitaires de France 10.3917/puf.gasp.2008.01

[B5] Gilligan C. (2009). Le care, éthique féminine ou éthique féministe? Multitudes 37–38, 76–78. 10.3917/mult.037.0076

[B6] Golay D. Malatesta D. (2012). L'amitié entre filles de 9 à 11 ans : entre affinités individuelles et enjeux statutaires. SociologieS. Available online at: http://sociologies.revues.org/4089

[B7] Lamprecht M. Bürgi R. Gebert A. Stamm H. (2017). Clubs sportifs en Suisse. Evolutions, défis et Perspectives. Macolin: OFSPO.

[B8] Laugier S. (2009). L'éthique comme politique de l'ordinaire. Multitudes 37–38, 80–88. 10.3917/mult.037.0080

[B9] Malatesta D. Jaccoud C. Golay D. (2014). Des publics juvéniles fabricants de cultures sportives : Le cas de deux sports pratiqués en club par des filles en Suisse romande. Agora débats/jeunesses 68, 113–126. 10.3917/agora.068.0113

[B10] Marston Tallec K. (2015). “A lost legacy of fraternity? The Case of European youth football,” in Routledge Handbook of Sport and Legacy. Meeting the Challenge of Major Sports Events. Holt R. Ruta D Ed (London: Routledge International Handbooks), 176–188. 10.4324/9780203132562-14

[B11] Modak M. (2015). Pascale Molinier: Le travail du care. Nouvelles Questions Féministes 34, 126–130. 10.3917/nqf.341.0126

[B12] Molinier P. (2013). Le travail du Care. Paris: La Dispute.

[B13] Murray S. B. (1996). “WE ALL LOVE CHARLES”: men in child care and the social construction of gender. Gender Society 10, 368–385. 10.1177/089124396010004002

[B14] Negura L. (2006). L'analyse de contenu dans l'étude des représentations sociales. SociologieS. Available online at: http://journals.openedition.org/sociologies/993

[B15] Paillé P. Mucchielli A. (2012). “Chapitre 11 - L'analyse thématique,” in L'analyse Qualitative en Sciences Humaines et Sociales. Paillé P Mucchielli A eds. (Paris: Armand Colin), 231–314. 10.3917/arco.paill.2012.01

[B16] Pantaléon N. (2003). Socialisation par les activités sportives et jeunes en difficultés sociales. Empan 3, 51–53. 10.3917/empa.051.0051

[B17] Parlebas P. (1999). Jeux, sports et sociétés. Paris: INSEP. 10.4000/books.insep.1067

[B18] Radway J. (1984). Reading the Romance. Women, Patriarchy and Popular Literature. Chapel Hill: The University of North Carolina Press.

[B19] Skeggs B. (2015). Des Femmes Respectables. Classe et Genre en Milieu Populaire. Marseille: Agone 10.3917/agon.berve.2015.01

[B20] Sorignet P. (2006). Danser au-delà de la douleur. Actes Recherche Sciences Sociales 63, 46–61. 10.3917/arss.163.0046

[B21] Tronto J. (2009). Un Monde Vulnérable. Pour Une Politique du Care. Paris: La Découverte.

